# Molecular characterization of Vibrio cholerae O1 isolates obtained from outbreaks in the Philippines, 2015–2016

**DOI:** 10.1099/jmm.0.001443

**Published:** 2021-11-24

**Authors:** Mark Philip Bugayong, Hidemasa Izumiya, Josie M. Bilar, Masatomo Morita, Eiji Arakawa, Mariko Saito-Obata, Hitoshi Oshitani, Makoto Ohnishi

**Affiliations:** 1 Department of Microbiology, Research Institute for Tropical Medicine, Muntinlupa, Philippines; 2 Department of Bacteriology I, National Institute of Infectious Diseases, Tokyo, Japan; 3 Department of Virology, Tohoku University Graduate School of Medicine, Miyagi, Japan; 4 RITM-Tohoku Collaborating Research Center for Emerging and Re-emerging Infectious Diseases, Muntinlupa, Philippines

**Keywords:** *V. cholerae *O1, Philippines, multilocus variable-number tandem repeat analysis, large-chromosome types, eBURST grouping, molecular epidemiology

## Abstract

**Introduction.** The Philippines, comprising three island groups, namely, Luzon, Visayas and Mindanao, experienced an increase in cholera outbreaks in 2016. Previous studies have shown that *Vibrio cholerae* isolates obtained from the Philippines are novel hybrid El Tor strains that have evolved in the country and are clearly distinct from those found in Mozambique and Cameroon.

**Gap statement.** The characterization of the strains isolated from outbreaks has been limited to phenotypic characteristics, such as biochemical and serological characteristics, in most previous studies.

**Aim.** We performed multilocus variable-number tandem repeat (VNTR) analysis (MLVA) for *V. cholerae* isolates obtained from 2015 to 2016 to further characterize and understand the emergence and dissemination of the strains in the Philippines.

**Methodology.** A total of 139 *V*. *cholerae* O1 Ogawa biotype El Tor isolates were obtained from the Philippines during diarrhoeal outbreaks in 18 provinces between 2015 and 2016. VNTR data were analysed to classify the MLVA profiles where the large-chromosome types (LCTs) were applied for grouping.

**Results.** We identified 50 MLVA types among 139 isolates originating from 18 provinces, and 14 LCTs. The distribution of the LCTs was variable, and a few were located in specific areas or even in specific provinces. Based on eBURST analysis, 99 isolates with 7 LCTs and 32 MLVA types belonged to 1 group, suggesting that they were related to each other. LCT A was predominant (*n*=67) and was isolated from Luzon and Visayas. LCT A had 14 MLVA types; however, it mostly emerged during a single quarter of a year. Eight clusters were identified, each of which involved specific MLVA type(s). The largest cluster involved 23 isolates showing 3 MLVA types, 21 of which were MLVA type A-14 isolated from Negros Occidental during quarter 4 of 2016. Comparative analysis showed that almost all isolates from the Philippines were distinct from those in other countries.

**Conclusions.** The genotypic relationship of the *V. cholerae* isolates obtained during outbreaks in the Philippines was studied, and their emergence and dissemination were elucidated. MLVA revealed the short-term dynamics of *V. cholerae* genotypes in the Philippines.

## Introduction

Cholera is an acute diarrhoeal disease caused by toxigenic *Vibrio cholerae* O1/O139 strains [1]. It is an important public health issue in endemic countries where access to safe water and adequate sanitation is limited. The global burden of cholera is estimated at 2.9 million cases occurring annually in 69 endemic countries with 95 000 deaths [2]. Multiple global pandemics of cholera have manifested since 1817. The seventh pandemic began in 1961 in Indonesia and is still ongoing, with a global spread [1]. *V. cholerae* O1 biotype El Tor is responsible for the current global spread of cholera that originated in the Bay of Bengal in South Asia [3]. Phylogenetic studies have identified sublineages and transmission of the agent in Africa, Asia, and Latin America [3,6].

The Philippines is a cholera-endemic country [2,7]. From 2008 to 2013, 42 071 suspected and confirmed cases were reported. The average annual incidence from 2010 to 2013 was 9.1 cases per 100000 individuals [7]. Cholera outbreaks in the country have occurred successively. The strains isolated from these outbreaks generally belong to serogroup O1 (Ogawa), biotype El Tor. Strain characterization has been limited to phenotypic characteristics, such as biochemical and serological characteristics. Only a few studies have been performed to further characterize *V. cholerae* isolates obtained in the Philippines. One study showed that the isolates from the Philippines were novel hybrid El Tor strains that had evolved in the country [8]. Another study showed that *V. cholerae* strains in the Philippines were clearly distinct from those found in Mozambique and Cameroon [9].

In 2016, there was an upsurge in cholera cases in several areas of the Philippines, which affected all three island groups: Luzon, Visayas and Mindanao. Molecular typing methods can provide insights into the relationships between causative agents to elucidate the mechanism underlying the emergence and dissemination of cholera in the Philippines. Multilocus variable-number tandem repeat (VNTR) analysis (MLVA) is a technique with high discriminatory power to distinguish between isolates [10,11].

In the present study, we performed MLVA-based molecular typing of *V. cholerae* O1 (Ogawa) El Tor isolates obtained from different areas of the Philippines that experienced cholera outbreaks between 2015 and 2016, aiming to further characterize and understand the emergence and dissemination of the strains in the Philippines.

## Methods

### Bacterial isolates

A total of 139 *ctx* (encoding cholera toxin)-positive *V. cholerae* O1 Ogawa biotype El Tor isolates obtained from cholera outbreaks in 18 provinces between 2015 (*n*=21) and 2016 (*n*=118) were used. These provinces are situated in Luzon, Visayas and Mindanao island groups.

### MLVA

MLVA was performed according to a procedure described previously [12]. Seven loci, VC-1, 2, 3, 5, 6, 7 and 8, corresponding to VC0147, VC0436-7, VC0500, VC1457-8, VC1650, VCA0171 and VCA0283 of *V. cholerae* N16961, respectively, were used. VC-1, 2, 3, 5 and 6 are present on the large chromosome of *V. cholerae*, and VC-7 and 8 are present on the small chromosome. The primer sequences used are shown in Table S1 (available in the online version of this article). Details regarding the loci and polymerase chain reaction (PCR) primers used for amplification have been published previously [13]. Total DNA was extracted using the QIAmp DNA Mini Kit (Qiagen GmbH, Hilden, Germany) according to the manufacturer’s instructions. The extracted DNA (approximately 50 ng) was used as the template for PCR amplification in a 15 µl reaction prepared using the Qiagen multiplex PCR Plus kit (Qiagen). Reaction conditions were as follows: 95 °C for 5 min; 35 cycles of 95 °C for 20 s, 60 °C for 90 s and 72 °C for 60 s; and 72 °C for 10 min. PCR products were separated with an ABI 3130xl Genetic Analyzer (Thermo Fisher Scientific, Tokyo, Japan). The band sizes were determined using GeneMapper software (Thermo Fisher Scientific) and converted to a repeat copy number. The null allele (no amplified product detected) was designated as −2, which is applied to the standardized data analysis [14,15].

### Data analysis

MLVA results were analysed using BioNumerics v.6.6 software (Applied Maths, Sint-Martens-Latem, Belgium). Simpson’s diversity index (D) was calculated according to a previously described formula [16]. A minimum spanning tree was created via the MST algorithm with a categorical coefficient using BioNumerics software. Because the two small chromosomal loci are highly variable [10,17], the MLVA types were designated as a combination of allele profiles of the five large chromosome-derived loci (MLVA5) [18] and all seven loci (MLVA7), such as A-01. The former types are called large-chromosome types (LCTs) here. An unrooted tree was created using a categorical coefficient and the unweighted pair group method with arithmetic mean (UPGMA) clustering with BioNumerics software v6.6. The eBURST group was assessed using goeBURST (http://www.phyloviz.net/goeburst/). In this study, ‘related’ and ‘closely related’ refer to single locus variation and single locus variation with one repeat difference, respectively. The assembled data from whole-genome sequencing in other studies were downloaded from the National Center for Biotechnology Information (NCBI) and subjected to *in silico* PCR with the primer sequences listed in Table S1 to extract those corresponding to the MLVA loci. The extracted sequences were converted to repeat copy numbers and were analysed in BioNumerics.

### Cartography

The maps were created using R version 3.5.2 (https://cran.r-project.org/) via the package maptools. Map data were obtained from an open-access site, the Database of Global Administrative Areas (https://gadm.org/index.html).

## Results and discussion

### MLVA type

We performed MLVA on 139 clinical *V. cholerae* isolates obtained from the Philippines. The discriminatory power of each VNTR locus is presented in Table 1. VC-3 and VC-5 showed D values of approximately 0. The highest D value calculated was 0.953 for VC-1, followed by 0.801 for VC-7 and 0.738 for VC-8. The highest number of identified alleles was 14 in VC-7, followed by 8 in VC-8 and 7 in VC-1.

**Table 1.** Discriminatory parameters of MLVA

Locus/type	Locus tag of N16961	No. of alleles	D*
VC-1	VC0147	7	0.953
VC-2	VC0436-7	3	0.617
VC-3	VC0500	1	0
VC-5	VC1457-8	2	0.07
VC-6	VC1650	5	0.358
VC-7	VCA0171	14	0.801
VC-8	VCA0283	8	0.751
LCT†		14	0.738
MLVA type		50	0.953

*Simpson’s diversity index.

†Large-chromosome type.

A total of 50 MLVA types were identified with a D value of 0.953 (Table 1). The MLVA profiles are shown in Table S2. Because the two VNTR loci in the small chromosome are hypervariable, grouping of MLVA results based on those of the large chromosome was applied [10,17, 18]. We identified 14 types based on the VNTR loci of the large chromosome (large chromosome type, LCT). The LCT-based D value was 0.738.

**Fig. 1.**
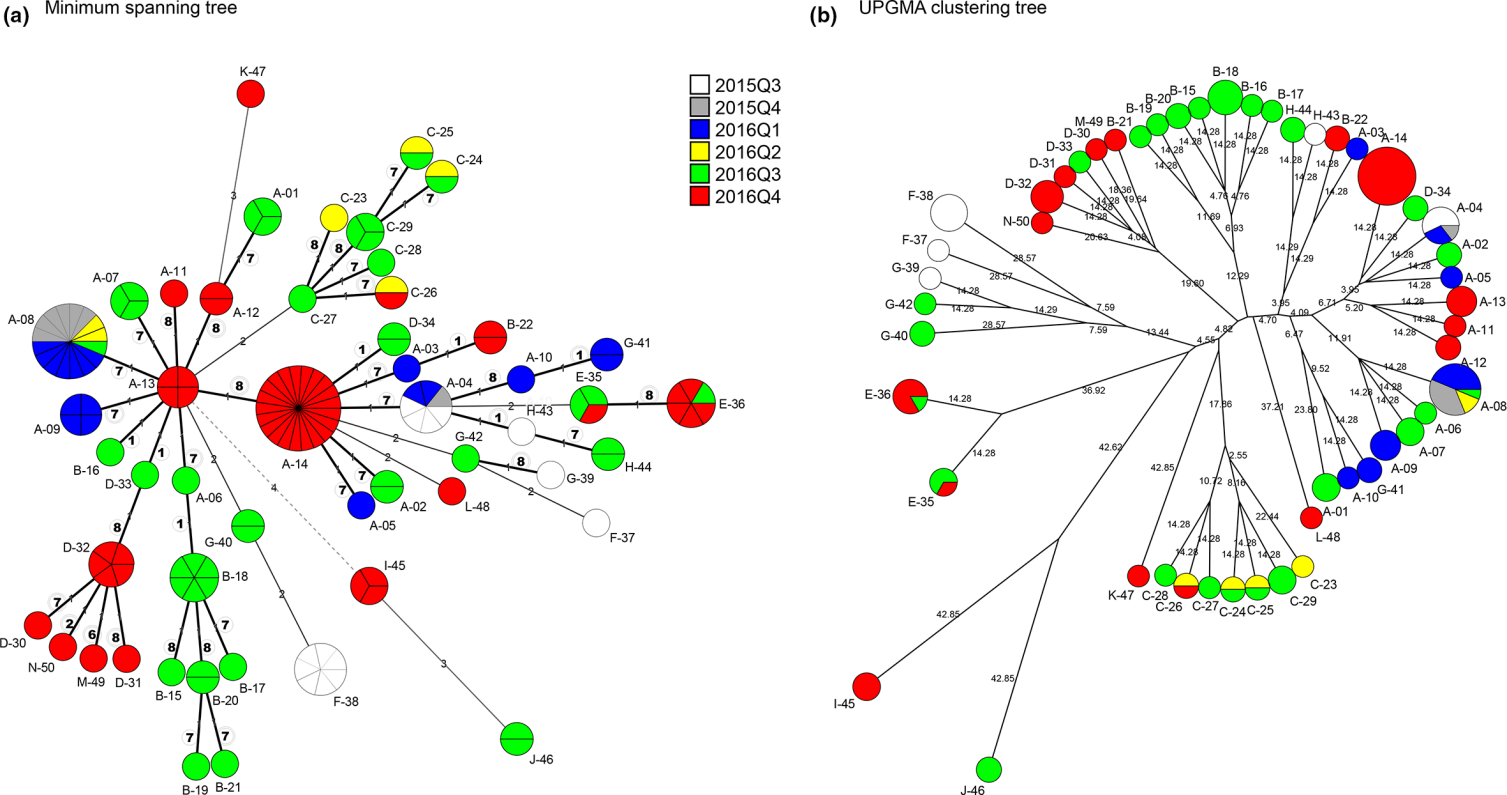
Network tree of the MLVA results. Each MLVA type is represented by a node with MLVA types. Size of the nodes corresponds to the number of isolates. The colours indicate the isolation periods. Those from 2015 are also hatched. Branch lengths are shown on the branches. (**a**) Minimum spanning tree. Thickness and length of the branches indicate the number of variable loci. Numbers in circles indicate VNTR loci (for example, ‘7’ means ‘VC-7’) linking the corresponding branches. Double circles indicate that the links are due to one repeat difference. (**b**) A network tree constructed using the categorical coefficient and UPGMA clustering of MLVA results. MLVA, multilocus variable-number tandem repeat analysis.

The minimum spanning tree showed that most of the isolates were linked to each other by single-locus variations (Fig. 1a). There were 39 links of single-locus variations in MST (Fig. 1a). Among them, seven, one and one links were due to the differences in VC-1, VC-2 and VC-6, respectively. Meanwhile, 18 and 12 links were due to VC-7 and VC-8, respectively, indicating that most links were accounted for by variations in the small chromosomal loci. The small chromosomal loci are of the coding regions. VC-7 and VC-8 encode VWA domain-containing protein and hypothetical protein, respectively. A previous study on VNTR mutation in *Escherichia coli* showed that the greater the allele number, the greater the mutation rate [19]. In this study, the repeat copy numbers were greater in VC-7/8 than in the other loci, which could result in hypervariability.

eBURST analysis identified 11 eBURST groups (eBG), with 3 singletons. Most of the isolates (*n*=99) were components of a large eBG1 that included 7 LCTs [A, B, D, H, M, and N and a part of LCT G (G-41)] and 32 MLVA types that differ from each other by a single locus, suggesting that they were related.

We also created an unrooted tree based on UPGMA clustering (Fig. 1b). It was observed that most of the isolates could be classified according to LCTs. A few discrepancies were observed between MST and UPGMA clustering. However, UPGMA clustering occasionally fitted to the epidemiology more than MST did, as described below. Thus, the former could complement the latter.

The characteristics of each LCT are described in the following sections.

### LCT A of eBG1

**Fig. 2.**
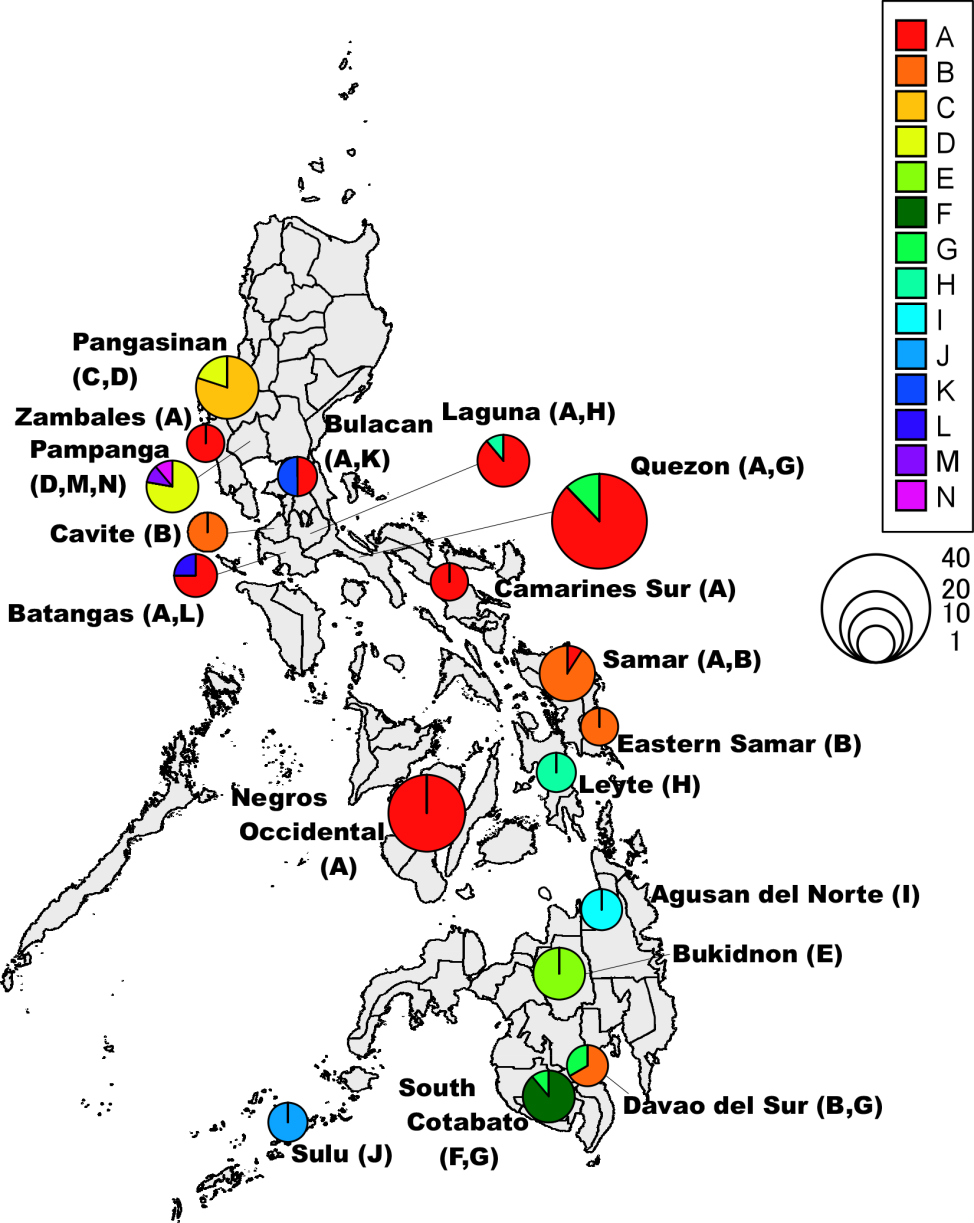
Geographical distribution of LCTs. The size and colours of pie charts correspond to the number of isolates and proportion of LCTs, respectively. Names of provinces and LCT in parenthesis are indicated. LCT, large-chromosome type.

**Fig. 3.**
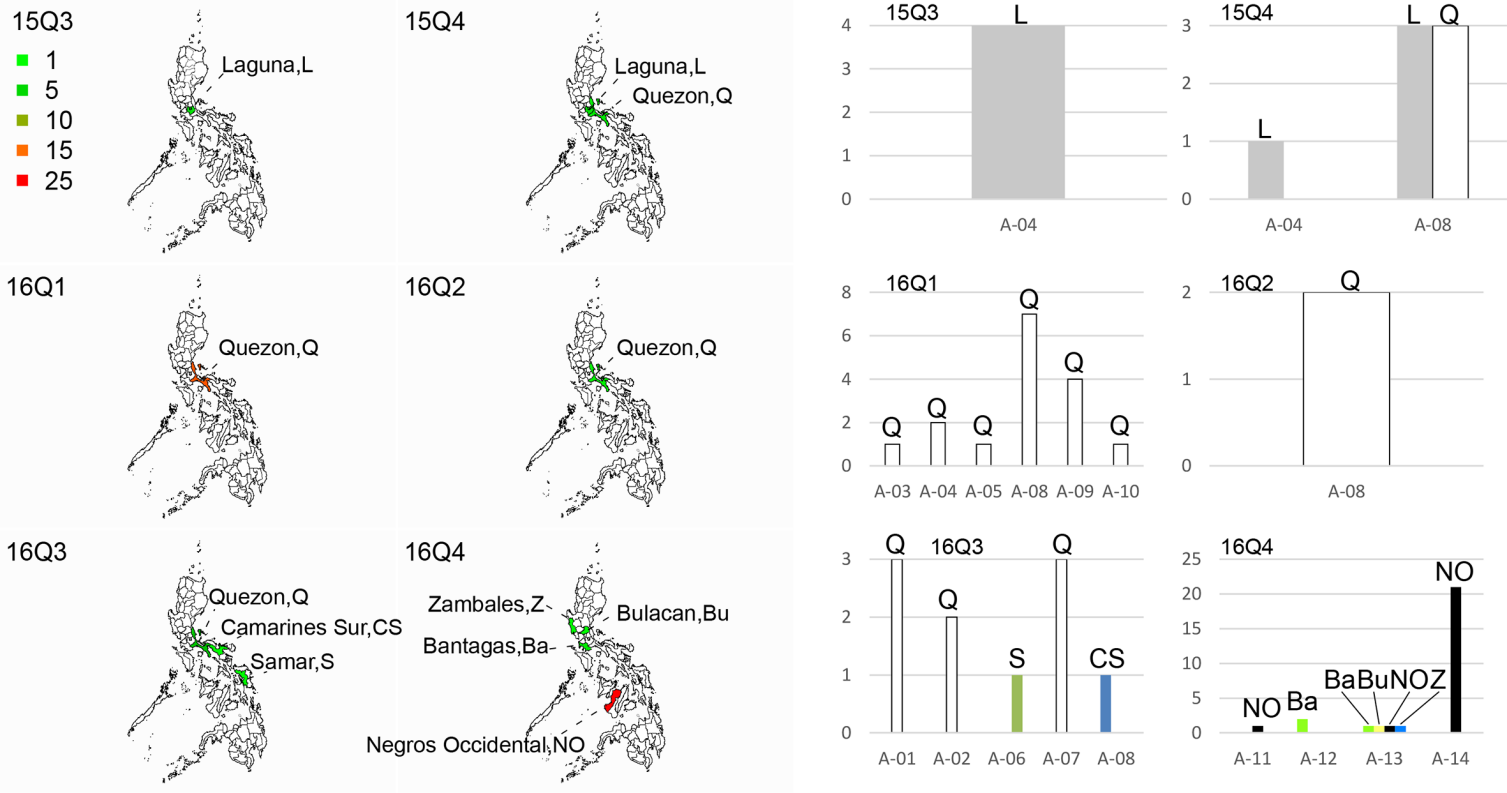
Distribution of LCT A isolates. The panels on the left show geographical distribution according to the isolation periods. Names of provinces and abbreviations are indicated. The panels on the right show the distribution of the MLVA types according to the isolation periods. Abbreviations for the province of isolation are stated above each bar. L, Laguna; Q, Quezon; S, Samar; CS, Camarines Sur; NO, Negros Occidental; Ba, Batangas; Bu, Bulacan; Z, Zambales; LCT, large-chromosome type; MLVA, multilocus variable-number tandem repeat analysis.

LCT A, which comprised 67 isolates with 14 MLVA types, was the most prevalent in eBG1. This LCT was dominant in Luzon (Zambales, Bulacan, Batangas, Quezon and Camarines Sur) and Visayas (Negros Occidental and Samar) (Fig. 2). In the LCT A group, the types A-11, A-13 and A-14 were only isolated from Visayas (Negros Occidental) (Table 2, Fig. 3). The predominant type, A-14 (*n*=21), was only identified in strains isolated during quarter 4 of 2016 (2016Q4). The second most dominant type, A-08 (*n*=16), emerged in Luzon (Laguna, Quezon and Camarines Sur) between 2015Q4 and 2016Q3 (Table 2, Fig. 3). The third most dominant type, A-04 (*n*=7), was isolated from Laguna and Quezon in 2015Q3–Q4 and 2016Q1, respectively (Table 2, Fig. 3).

**Table 2.** Distribution of MLVA types of eBG1 isolates

**Year/quarter**	**2015/Q3**	**2015/Q4**		**2016/Q1**	**2016/Q2**	**2016/Q3**							**2016/Q4**						
				**LUZON**						**VISAYAS**		**MINDANAO**			**LUZON**			**VISAYAS**	Total
MLVA type	Laguna	Laguna	Quezon	Quezon	Quezon	Pangasinan	Quezon	Camarines Sur	Eastern Samar	Leyte	Samar	Davao del Sur	Batangas	Bulacan	Cavite	Pampanga	Zambales	Negros Occidental	
A-01							3												3
A-02							2												2
A-03				1															1
A-04	4	1		2															7
A-05				1															1
A-06											1								1
A-07							3												3
A-08		3	3	7	2			1											16
A-09				4															4
A-10				1															1
A-11																		1	1
A-12													2						2
A-13													1	1			1	1	4
A-14																		21	21
B-15											1								1
B-16												1							1
B-17											1								1
B-18									1		4	1							6
B-19											1								1
B-20											2								2
B-21											1								1
B-22															2				2
D-30																1			1
D-31																1			1
D-32																5			5
D-33						1													1
D-34						2													2
G-41				2															2
H-43	1																		1
H-44										2									2
M-49																1			1
N-50																1			1
**Total**	5	4	3	16	2	3	8	1	1	2	11	2	3	1	2	9	1	23	97

It is likely that A-14 was clustered together with A-11 and A-13, as they differed by a single locus, VC-8, and it caused an outbreak in Negros Occidental in 2016Q4. Other isolates with A-12 and A-13 from Luzon (Zambales, Bulacan and Batangas) in 2016Q4 were also single-locus variants of A-14, but the epidemiological relationship was not uncovered.

A-04 and A-08 differed by a single locus with one repeat in VC-8, and overlapped with each other in the same area. A-04 disseminated prior to A-08. Although A-08 was dominant in 2016Q1, several minor variants were also isolated from Quezon (Fig. 3), where they differed from each other by a single locus, VC-7 or VC-8. Furthermore, G-41, a component of eBG1, was isolated from Quezon during 2016Q1. G-41 was a single-locus variant (by one repeat) of A-10 (Fig. 1a) in VC-1. Therefore, closely related isolates, such as A-04, A-08 and G-41, could have evolved in the area by developing variants. Although A-08 spanned most of the study period (2015Q4–2016Q4), the majority (12 of 14) of MLVA types emerged during a single quarter of a year. This was the same in MLVA types of other LCTs, suggesting the short-term dynamics of *V. cholerae* genotypes.

### LCT B of eBG1

LCT B had 8 MLVA types present in 15 isolates (Table 2). LCT B was isolated from Luzon (Cavite), Visayas (Samar and Eastern Samar) and Mindanao (Davao del Sur) (Fig. 2). LCT B differed from LCT A by three repeats in VC-1. The distribution of LCT B was apparently distinct from that of LCT A (Fig. 2). With the exception of B-22, almost all LCT B isolates obtained during 2016Q3 were clustered in Samar and Eastern Samar (Figs1a  2). MLVA type B-22 alone was isolated during 2016Q4 and accounted for the isolates in the northern province of Cavite (Table 2). B-22 was assigned to a different branch in MST because it differed by two loci against any of the other LCT B isolates and differed by a single locus, VC-1, with three repeats against A-03 (Fig. 1a).

Based on network analysis, B-16 was assigned next to A-13 in MST because of a single locus variation in loci VC-1 by three repeats. B-16 was also a single locus variant of B-18 in VC-7 by one repeat, leading to an assignment to the near branches in Fig. 1b. The geographical closeness of B-16 and one of the B-18 isolates, as both were isolated in Davao del Sur, could support their genetic closeness (Table 2). However, the relationship between LCT B isolates from Samar/Eastern Samar and Davao del Sur was not revealed because of limited epidemiological information.

### Other LCTs of eBG1

LCT D had 5 MLVA types present in 10 isolates. LCT D was isolated from the northern area (Pampanga and Pangasinan in Luzon) (Fig. 2). The MLVA types, D-33 and D-34, were only isolated from Pangasinan during 2016Q3 (Table 2). Although D-33 and D-34 were placed in different branches in the MST (Fig. 1a), D-34 was a single-locus variant of D-33 in VC-8 by one repeat as well as of A-14 by four repeats. The MLVA types, D-30, D-31 and D-32, were clustered in both trees and were only isolated from Pampanga during 2016Q4. LCTs M and N were also included in the cluster of LCT D (Fig. 1b). They differed from each other by a single locus (VC-2, VC-6, VC-7, or VC-8) with one repeat, and were only isolated from Pampanga during 2016Q4 (Fig. 2). Furthermore, D-33/D-34 differed from D-30/D-31/D-32 by at least two repeats. Therefore, the isolates of D-33 and D-34 and those of D-30, D-31, D-32, M-49 and N-50 could have created two distinct clusters, respectively.

LCT H had two MLVA types present in three isolates. H-43 and H-44 were clustered in both the trees and differed by three repeats in VC-7. Isolates of each MLVA type were isolated from different areas and periods: H-43 from Laguna during 2015Q3 and H-44 from Leyte during 2016Q3 (Table 2).

### Additional eBURST groups

In addition to the large eBURST group (eBG1), each LCT created an individual eBURST group (Table 3). LCT C had 7 MLVA types present in 12 isolates. LCT C was only isolated from the province of Pangasinan in Luzon during 2016Q2–Q4 (Table 3). LCT C isolates differed from each other by one repeat in a single locus, suggesting a possible circulation of the closely related clones in a limited area.

**Table 3.** Distribution of MLVA types other than eBG1 isolates

Year/quarter		2015Q3	2016Q1	2016Q2			2016Q3					2016Q4			
		**Mindanao**		**Luzon**				**Mindanao**			**Luzon**		Mindanao		Total
MLVA type	*eBG*	South Cotabato	Quezon	Pangasinan	Pangasinan	Quezon	Bukidnon	Davao del Sur	Sulu	Batangas	Bulacan	Pangasinan	Agusan del Norte	Bukidnon	
C-23	2			1											1
C-24	2			1	1										2
C-25	2			1	1										2
C-26	2			1								1			2
C-27	2				1										1
C-28	2				1										1
C-29	2				3										3
E-35	3						2							1	3
E-36	3						1							5	6
F-37	5	1													1
F-38	6	7													7
G-39	4	1													1
G-40	7					2									2
G-42	4							1							1
I-45	8												3		3
J-46	9								2						2
K-47	10										1				1
L-48	11									1					1
**Total**		9	2	4	7	2	3	1	2	1	1	1	3	6	42

LCT E had two MLVA types present in nine isolates and was only isolated from the province of Bukidon in Mindanao between 2016 Q3 and Q4.

LCT F had two MLVA types present in eight isolates and was only isolated from the province of South Cotabato in Mindanao during 2015Q4.

LCTs I, J, K and L showed long branches in both trees and were isolated from Agusan del Norte during 2016Q4, from Sulu during 2016Q3, from Bulacan during 2016Q4 and from Batangas during 2016Q4, respectively.

LCT G had four MLVA types present in six isolates. G-41 was included in eBG1 and was isolated from Quezon during 2016Q1, in addition to LCT A isolates. G-39 and G-42 were single-locus variants in VC-8 with a one-repeat difference, whereas the others were double-locus variants. Each MLVA type of LCT G was isolated spatially and temporally in a distinct manner: G-39 from South Cotabato (2015Q3), G-40 from Quezon (2016Q3) and G-42 from Davao del Sur (2016Q3) (Table 3).

### Comparative analysis

We identified eight clusters in this study: (1) 2016Q4 Negros Occidental (representative MLVA type, A-14); (2) 2016Q4 Cavite (B-22); (3) 2016Q3 Samar/Eastern Samar (B-18); (4) 2016Q4 Pampanga (D-32); (5) 2016Q3 Pangasinan (D-34); (6) 2016Q3 Leyte (H-44); (7) 2016Q4 Agusan del Norte (I-45); and (8) 2016Q3 Sulu (J-46). Each cluster was genotypically and spatiotemporally supported. Among the clusters, those belonging to eBG1 were single-locus variants for each other and could make a clonal complex. Analyses in this study uncovered predominant and minor complexes. However, the location of the isolates in this study against those of other countries are not clear.

A previous study showed that the *V. cholerae* strains that caused an outbreak in the Palawan, West of Negros Occidental in 2011 were located within the seventh pandemic phylogeny but were distinct from recent isolates from other countries [8]. We compared the MLVA profiles in this study with previous studies on genomics of *V. cholerae*, including those on the epidemics in Africa, Asia and Latin America [4,6,20].

A total of 228 data from the previous studies were included, the metadata of which are shown in Table S3. The MST in Fig. S1 shows that almost all the isolates in this study formed a large group, distinct from those in other areas of the world. The reference strain N16961 was located in the central part of the MST that differed by three loci from the nearest MLVA profile. A few isolates from the Philippines in the 1960s–1970s [5] were located near the centre of the MST (Fig. S1, 1960sPHL and 1973PHL). The 1960sPHL shared an MLVA profile (8, 6, 4, 4, 6, 10, 9) with isolates of Egypt, Vietnam, Pakistan and New Guinea, all of which were from the 1960s. A Haitian strain in 2010 (2010Haiti) [22] was located in the central part and could form a complex with isolates from South Asia. The Yemeni strains (2017Yemen) [6] formed a complex in the bottom part of the MST. The epidemic strains from Southeast Asia in 2007–2010 [18,27,29] were located on an edge in the bottom part of the MST. All were distinct from the isolates in this study. The Palawan outbreak strains (2011PHL-a,b) consisted of two MLVA profiles (12, 7, 4, 4, 9, 18, 25) and (12, 7, 4, 4, 10, 14, 21), respectively. The former was located within the branches of the isolates in this study; between G-40 and I-45. By contrast, the latter was located in the bottom part of the MST. The two profiles being far apart could be attributed to the differences in three loci of the profiles. I-45 and J-46 in this study were the most out-grouped in Fig. 1. I-45 was still within the group of the Philippines (Fig. S1), which might be due to the link with 2011PHL-a by a double-locus variation. J-46 was located in the central part of the MST of Fig. S1. Considering that both 2011PHL-a,b belonged to the seventh pandemic phylogeny [8], all the isolates in this study would be a part of the seventh pandemic strains. Deeper analyses using whole-genome sequencing could reveal the phylogeny of the isolates more clearly.

Our study revealed the genetic relationships among *V. cholerae* isolates in the Philippines that were obtained from 2015 to 2016. Several genetic clusters were identified that are in accordance with the spatiotemporal terms. We also revealed the short-term dynamics of the MLVA types. A comparative analysis suggested that the strains from the Philippines could be distinctive from those of other parts of the world, although they seemed to be a part of the current seventh pandemic.

## supplementary material

10.1099/jmm.0.001443 Uncited Fig. S1.

10.1099/jmm.0.001443 Uncited Table S1.

## Abbreviations

D value: Simpson's diversity index
eBG: eBurst group
LCT: large-chromosome type
MLVA: multilocus variable-number tandem repeat analysis
MST: minimum spanning tree
UPGMA: unweighted pair group method with arithmetic mean
VNTR: variable-number tandem repeat
