# Supplementary figures and images for: Molecular characterization of Vibrio cholerae O1 isolates obtained from outbreaks in the Philippines, 2015–2016
Source: J Med Microbiol. 2021 Nov 24;70(11):001443. doi: 10.1099/jmm.0.001443 (PMC11251456; doi:10.1099/jmm.0.001443)

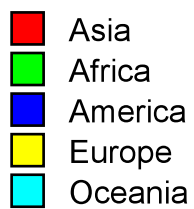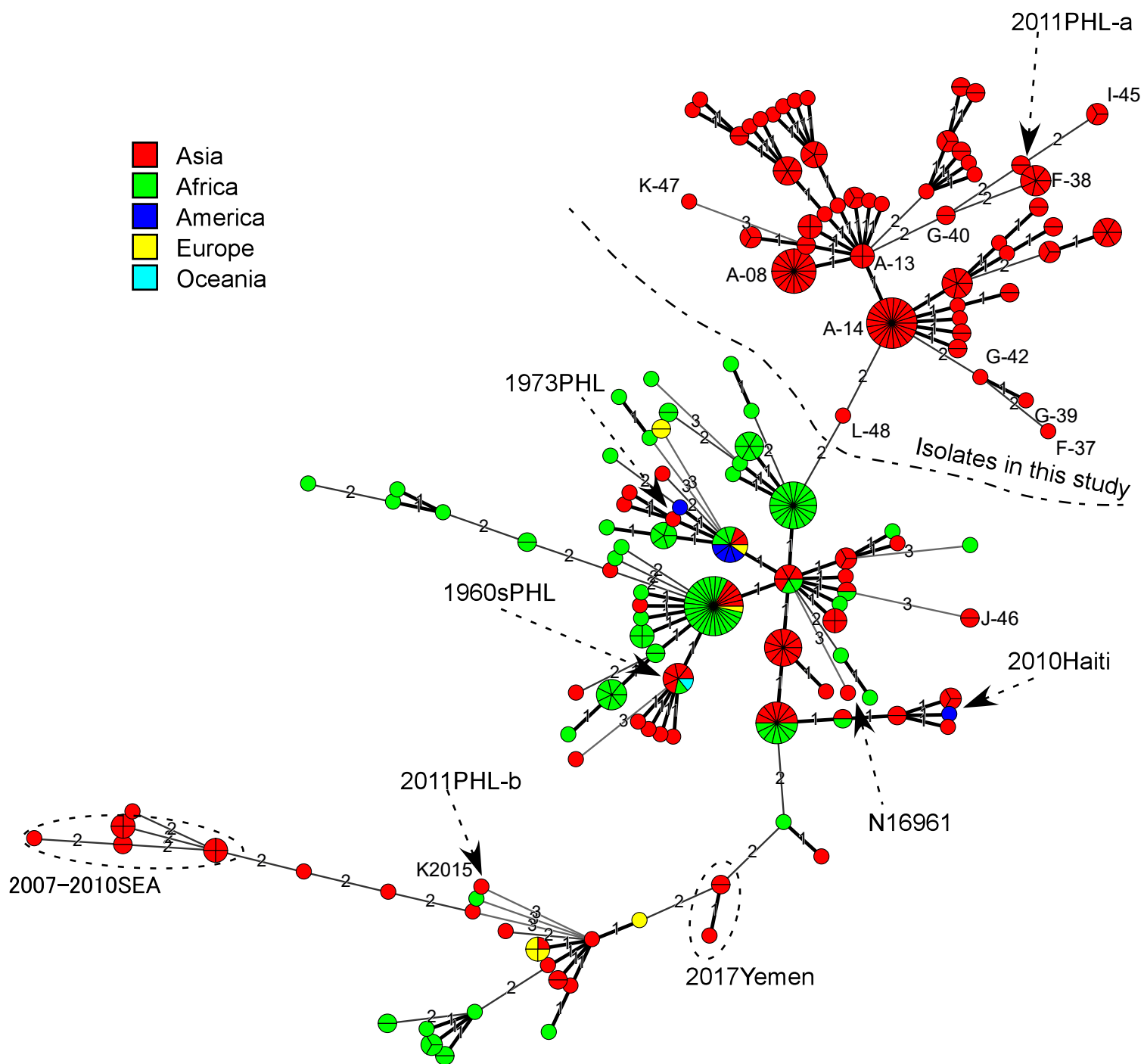

Supplement: Uncited Fig. S1. [file jmm-70-01443-s001.pdf]
